# Examining determinants of sexual behavior among indigenous adolescents in Taiwan

**DOI:** 10.1097/MD.0000000000015562

**Published:** 2019-05-13

**Authors:** Li-Mei Lin, Tao-Hsin Tung, Mei-Yu Yeh

**Affiliations:** a Department of Nursing, Chang Gung University of Science and Technology, Tao-Yuan; b Department of Medical Education and Research, Cheng-Hsin General Hospital, Taipei; c Department of Nursing, Mackay Medical College, New Taipei City, Taiwan.

**Keywords:** adolescents, indigenous, sexual behaviors

## Abstract

A recent Taiwanese government report indicated that there were an increased number of sexual encounters among 15- to 17-year-old adolescents. Among them, indigenous Taiwanese had a higher rate of sexual encounters than did the rest of the population. However, no prior study has surveyed indigenous Taiwanese adolescents’ sexual behavior. Therefore, we examined the determinants of sexual behavior among indigenous adolescents in Taiwan.

In this cross-sectional study, the researchers chose 16 high schools as the target research population; after 2-stage random sampling, 4 of the 16 high schools were recruited to participate in the research. Data were collected through a self-report questionnaire from the participants, with a total of 521 valid responses.

Of the indigenous adolescents surveyed, 53% (N = 275) said they had touching, hugging, or kissing experiences, while 15.7% (N = 82) reported sexual behavior. The higher rate of sexual encounters among indigenous Taiwanese adolescents is associated with the gender, number of current or past romantic partners, drinking, and drug use before sexual intercourse. After adjustment for confounding factors, gender (male vs female, adjusted OR = 3.33, 95% CI: 1.83–6.07), number of heterosexual (≧1 vs no, OR = 1.67, 95% CI: 1.36–2.06), and heterosexual relationship (yes vs no, OR = 3.81, 95% CI: 1.94–7.48) appeared to be statistically significantly related sexual behaviors.

This study found that the occurrence rate of sexual behavior for indigenous adolescents was higher than the past research results, and having initiated sexual intercourse in earlier age. The results also showed the indigenous adolescents whose drinking alcohol and drug use are more experiences on sexual behaviors. The results could be applied on sexual education program in campus.

## 1 Introduction

Teen unprotected sexual activity has become a major public health challenge. In 2017, the US Centers for Disease Control and Prevention conducted a national survey on adolescents aged 15 to 18 years old (i.e., students in grades 9–12), and found that 39.5% had had a sexual experience.^[1]^ Of that population, 37.7% were female and 41.4% were male; additionally, 28.7% were currently sexually active, 18.8% drank alcohol or did drug before sexual intercourse.^[1]^

Between 2011 and 2015, the US National Survey of Family Growth sampled 8.5 million adolescents aged 15 to 19 years old, and found that 16% of males and 11% of females had had sex for the first time under the age of 15 years. The percentage who had had sex increased to 68% for females and 69% for males by the age of 19 years.^[2]^ Moreover, the sexual experiences of adolescents in various US ethnic groups have been found to be significantly different.^[1–3]^ Sociocultural backgrounds are one probable explanation for the rate differences between ethnic groups.^[3–6]^ Notably, Black and Hispanic male adolescents have been determined to engage in a higher percentage of sexual behavior than the females of any ethnic group.^[1,2,5]^ For example, a 2017 US national survey of 15 to 19-year olds found that 52.7% of Black American male adolescents had had a sexual experience, a much higher occurrence rate of sexual experiences than that of White males (38.5%) and Hispanic males (44.1%).^[1]^ Additionally, a 2016 US National Center for Health Statistics report on birth rates among adolescents aged 15 to 19 years old revealed that the probability of birth rates was 3.9% among Asian adolescents, 29.3% among Black adolescents, 31.9% among Hispanic adolescents, 35.1% among American Indian and Alaska Native adolescents, and 14.3% among White adolescents.^[7]^

Shovellera et al^[6]^ estimated that about 10.2% of sexually active female adolescents became pregnant, and most of them had an abortion. 16.7% of male and 13.4% of female adolescents did not use a condom during sex. According to a 2015 report from Bureau of Health Promotion, Department of Health, 11.1% of the 5458 Taiwanese students in grades 10 to 12 had engaged in sexual behavior.^[8]^ The study also noted that of those students, 20.2% of male and 11.5% of female adolescents did not use a condom during sex.^[8]^ These results are worrisome, particularly the prevalence of unprotected sexual behavior and adolescent pregnancy.^[9]^

In Taiwan, a 2009 study demonstrated that more than 1% of interviewed adolescents took illegal drugs, such as opium, morphine, heroine, secobarbital, methaqualone, or cocaine, and that the percentage of males with this behavior was 2% higher than that of females. Moreover, the misuse of ecstasy or glue was higher than 2.5%.^[10]^ Many indigenous Taiwanese students in grades 10 to 12 were also determined to exhibit problem drinking at a rate 2.98 times higher than their Han counterparts.^[11]^ Other studies have shown that adolescents’ drinking or drug use increased the occurrence of risky sexual behavior.^[12–15]^ Currently, research on intimate or sexual behavior among indigenous Taiwanese adolescents is lacking. Therefore, this study is conducted to examine the determinants of sexual behavior among indigenous adolescents in Taiwan.

## 2 Methods

### 2.1 Research participants

Indigenous Taiwanese people represent 2.37% of the total population of Taiwan, and 98% of them live in eastern Taiwan;^[16]^ therefore, the researchers selected indigenous students from 16 high schools located in the Hualien and Taitung counties. A stratified random sampling method was adopted in which 4 schools were chosen at random. Each school had at least 80 indigenous students and 4 classes of students per grade from grades 10 to 12. The number of research participants from each school varied from 82 to 176.

The method of estimating the number of samples was applying the Sample Size Calculator to achieve confidence level 95% and confidence interval=3, with population, the minimum required size was estimated to be 516 subjects. A multistage sampling technique was used to select participants for the study. Four high schools identified as A, B, C, and D were randomly selected from the 16 high schools. The study population recruited from A, B, C, and D was 82, 122, 176, and 141 respectively in the proportion of 0.16: 0.23: 0.34: 0.27 among high schools.

### 2.2 Data collections

Data were collected through a cross-sectional survey of self-report questionnaires from the participants. The researchers eliminated 6 incomplete questionnaires, leaving a total of 521 valid responses. The research tool retrieved information on the participants’ demographic characteristics (e.g., gender, grade, ethnicity), family situation, intimate behavior (e.g., touching, hugging, or kissing other people), sexual behavior (e.g., having oral sex, vaginal intercourse, or anal intercourse with others), having a current romantic partner, and the number of former romantic partners. The participants also indicated whether they engaged in smoking, drinking alcohol, or drug use before sexual intercourse.

After being randomly selected, the sampled schools were informed; all of them officially agreed to participate in the survey. The research tool was distributed only after verification of the reliability and validity of the measures. The survey was completed during the participants’ regular school schedule. Because the content required that the participants’ personal privacy be ensured, a statement emphasizing the anonymous and voluntary nature of the survey was issued. To increase the validity of the questionnaire, the researcher and assistants went to the classrooms and conducted the entire procedure in person. After receiving an explanation of the research, all of the participants signed an agreement form and spent approximately 15 minutes completing the questionnaire.

### 2.3 Ethical considerations

This research project had reviewed and provided funding (No. 93-111024) by Chang Gung University of Science and Technology. Research materials had completed the collection in May 2004. The Ministry of Health and Welfare of Taiwan has enacted “The Human Subjects Research Act” since December 28, 2011. According to the regulation, all personal information should be protected in privacy. Besides that, if the minors are research objects of the research, their legal representative/guardian should be informed and need to obtain their consent as well.^[17]^ Since March 2017, another regulation of “The Human Subjects Research Act” had ruled that if research objects focus on indigenous people, a consultation meeting needs to be launched and obtained consent from the target group.^[17]^ Therefore, before December 28 of 2011, the indigenous related research only requires a statement that the subject must agree to the study in writing and may not expose personal privacy information, and does not mandate an IRB review before research execution.

### 2.4 Statistical analysis

All analyses were performed using SPSS 22.0. Descriptive statistics and a *χ*^2^ test were used to describe the participants’ gender and associated factors regarding their sexual and intimate experiences, and to elucidate differences between the participants who had and had not had a sexual experience. Multiple logistic regression was also performed to investigate the independence of risk factors associated with sexual behaviors. A *P* value of <.05 was considered to represent statistically significant difference.

## 3 Results

The participants (N = 521) were all indigenous Taiwanese and had an average age of 17.64 years. Table 1 presents a summary of their sociodemographic characteristics. Notably, 60.5% were female (N = 315) and 39.5% were male (N = 206), and 36.1% (N = 188), 34.5% (N = 180), and 29.4% (N = 153) were in grades 10, 11, and 12, respectively.

**Table 1:**
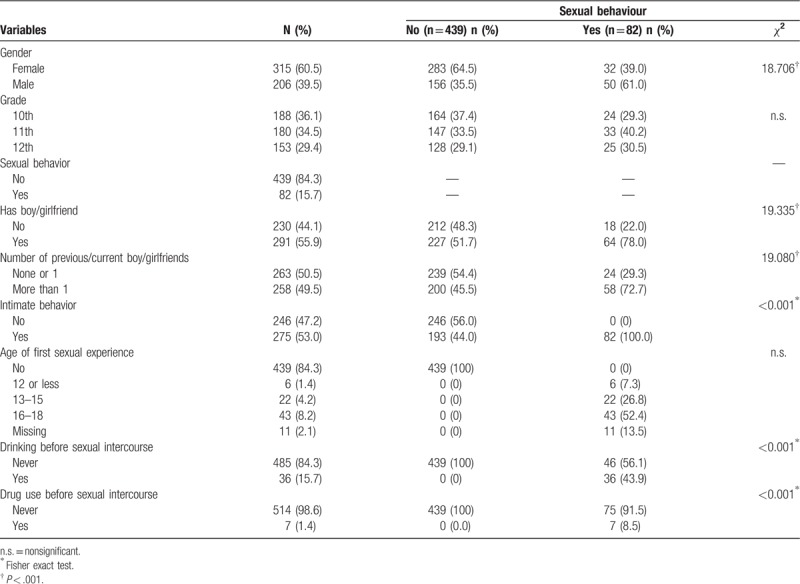
*χ*^2^and Fisher exact tests on the characteristics associated with sexual behaviors (n = 521).

Table 1 also displays the results of the *χ*^2^ and Fisher exact tests on the characteristics associated with sexual behavior. In total, 55.9% (N = 291) of the participants currently had a heterosexual partner at the time of the survey, and 49.5% (N = 258) had had more than 1 romantic partner during their lives (Table 1). More than half (53%, N = 275) had had intimate behaviors (touching, hugging, or kissing) and approximately 15.7% (N = 82) had had at least 1 sexual experience, which occurred on average between the ages of 16 and 18 years (8.2%, N = 43).

A few of the participants (4.2%, N = 22) had had their first sexual experience between the ages of 13- and 15-years old, and 6 of them (1.4%, N = 6) had had such an experience when they were 12-years old or younger. Among the 15.7% of participants who had already had a sexual experience, 43.9% (N = 36) had drunk alcohol and 8.5% (N = 7) had used drugs before engaging in sexual intercourse (Table 1). The results show that gender differences were evident in the sexual behavior of the participants; specifically, males’ sexual behavior was significantly higher than that of females. Those who had a current steady partner or who had had more than 1 partner had a higher likelihood of sexual behavior.

**Figure 1:**
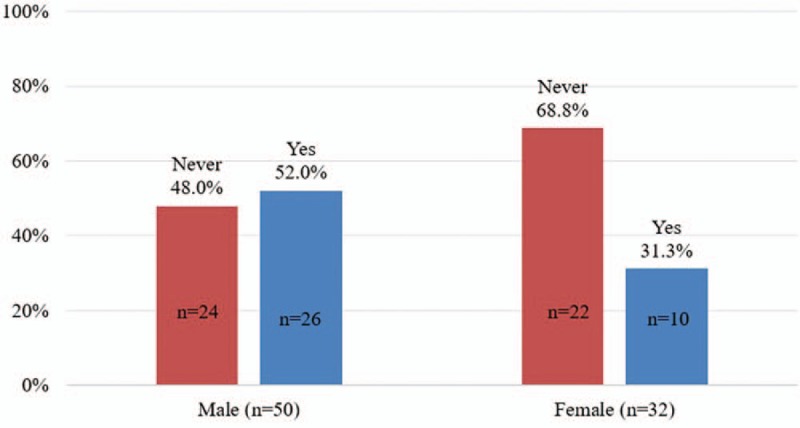
Drinking before sexual intercourses.

**Figure 2:**
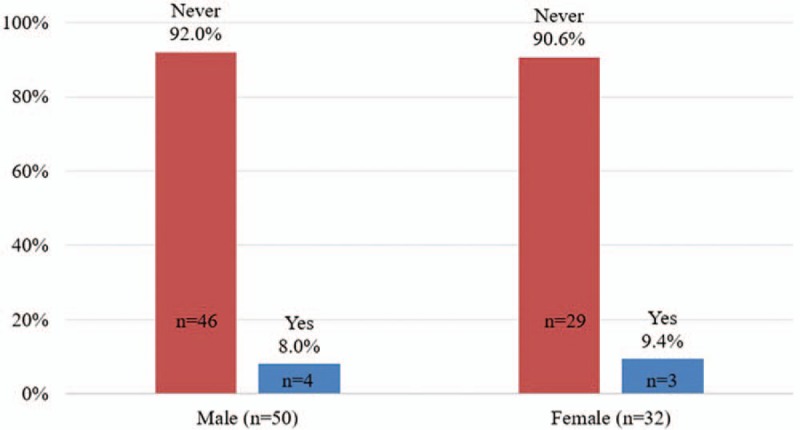
Drug before sexual intercourses.

**Figure 3:**
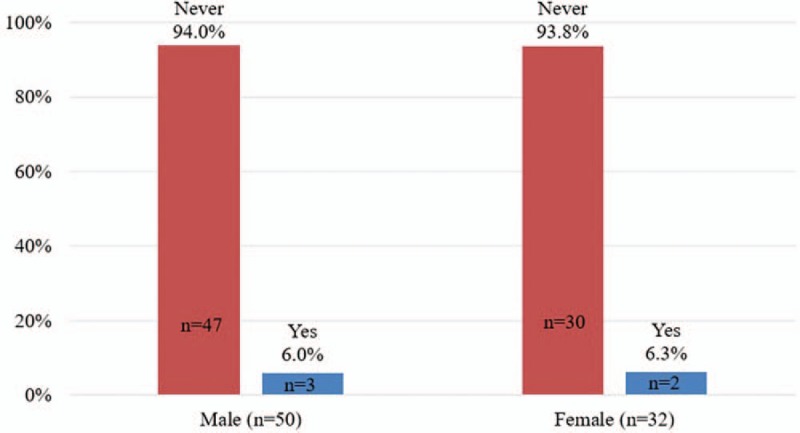
Drinking and drug before sexual intercourses.

Figure 1 shows that the prevalence of having alcohol before sexual intercourse was higher among males (52.0%, N = 26) than among females (31.25%, N = 10); the prevalence of using other drugs before sexual intercourse was higher among females (9.4%, N = 4) than among males (8.0%, N = 3) (Fig. 2). In Fig. 3, the prevalence of both alcohol and other drug use before sexual intercourse ranged from 6.0% to 6.25% (male = 3, female = 2).

The effects of independent associated factors upon sexual behaviors were examined using the logistic regression model. As is depicted in Table 2, gender (male vs female, adjusted OR = 3.33, 95% CI: 1.83–6.07), number of heterosexual (≧1 vs no, OR = 1.67, 95% CI: 1.36–2.06), and heterosexual relationship (yes vs no, OR = 3.81, 95% CI: 1.94–7.48) appeared to be statistically significantly related sexual behaviors.

**Table 2:**
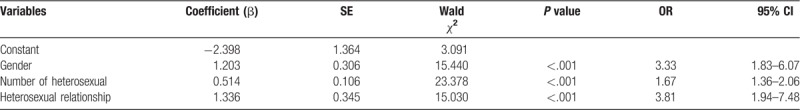
Logistic regression for sexual behaviors in indigenous adolescents.

## 4 Discussion

### 4.1 The practical implications

The results of this study found that 82 of the 521 (15.7%) indigenous adolescents had had at least 1 sexual experience, comprising 50 of the 206 male participants (24.3%) and 32 (10.2%) of the 315 female participants. According to a 2015 study conducted by Taiwan's Ministry of Health and Welfare concerning the general Taiwanese high-school student population, 11.8% of male students and 10.4% of female students self-reported having had at least 1 sexual experience.^[18]^ These results are higher than those of a similar 2012 survey that adopted the probability of proportional sampling method to study national high-school students in Taiwan, which found that the rate of self-reported sexual behavior was 7.7% for male students and 3.6% for female students.^[19]^

Our results indicate that the prevalence of sexual behavior among indigenous Taiwanese adolescents is higher than the national average reported in Taiwan. Also, the indigenous female adolescents are less likely to have sex than their Han counterparts, while males are more than twice as likely, compared with the 2015 study. However, no prior investigation has specifically considered indigenous Taiwanese adolescents’ sexual behavior, and also no meaningful comparison can be made until you resolve the massive difference in findings between the previous studies. Ramisetty-Mikler et al^[20]^ found that drinking and drugs are significant predictors of indigenous Hawaiian adolescent sexual behavior; approximately 25% of sexually active White and Indigenous Hawaiian females use alcohol or drugs before sexual behavior. In the present study, some of the sexually active participants (male: 43.9%, N = 36; female: 8.5%, N = 7) similarly reported using alcohol or drugs before engaging in sexual behavior.

Earlier literature has noted that culture affects the age at which sexual behavior first occurs.^[6,21]^ Rathus et al^[22]^ studied adolescents in 32 countries, and showed that sexual behavior was influenced by sociocultural, ethnic, gender, and racial differences. The present study focused on indigenous Taiwanese adolescents. The indigenous population of Taiwan comprises multiple ethnic groups, each of which has its own unique culture. The largest group, called Amis, are a matriarchal society. In Taiwan, indigenous families are traditionally very welcoming of guests; alcohol also plays an irreplaceable role in traditional harvest festivals, ancestral worship, and wedding ceremonies. Although selling liquor to minors is prohibited in Taiwan, 16.7% of Taiwanese adolescents drink at least once a month.^[23]^ In an examination of different ethnic groups, indigenous Taiwanese adolescents showed a higher prevalence rate of alcohol use than did their nonindigenous counterparts.^[11]^ Women are the center of the family network, and they practice matrilocal marriage (wherein the groom moves into the bride's house). From the age of 12 years onward, young Amis people participate in traditional ethnic activities, and males and females begin having close interactions at that time.^[24,25]^ Whether this is related to the occurrence of indigenous adolescents’ sexual behavior is unclear and should be studied further.

A 2015 sexual behavior survey of adolescents aged 15 to 19-years old in the United States showed that, by the age of 15, 68% of African-American male adolescents had had at least 1 sexual experience.^[1]^ Ramisetty-Mikler et al^[20]^ conducted a study among indigenous, White, and Asian adolescents in the Hawaii region and found that indigenous Hawaiian adolescents had a higher sexual behavior rate than did their White and Asian counterparts; the age at which their first sexual experience occurred was also earlier for the indigenous Hawaiian adolescents than for the other ethnic groups. A comparison of adolescent sexual behavior among indigenous Hawaiian, White, Filipino, and Japanese adolescents found that indigenous Hawaiian adolescents had the highest prevalence of sexual behavior (Hawaiian 46.5%, White 32.9%, Filipino 32.2%, and Japanese 19.9%). Additionally, more of the indigenous Hawaiian adolescents (9.7%) had their first sexual experience before they were 13-years old than did the White (4.5%), Filipino (3.1%), or Japanese (1.3%) adolescents. A higher percentage of the indigenous Hawaiians also had 4 or more sexual partners (Hawaiian 12.7%, White 10.9%, Filipino 3.1%, and Japanese 1.3%).^[26]^

Drinking and drug use can increase sexual desire and improve the pleasure of sex, and research has suggested that adolescent drinking or drug use significantly increases the incidence of sexual behavior and risky sexual behavior.^[27–29]^ The effects of alcohol and drug use on adolescent sexual behavior are a major concern because they reduce attention to safe sex practices, with the possible consequences of sexually transmitted diseases or accidental pregnancy.^[30,31]^

### 4.2 Methodological considerations

Several limitations should be considered when interpreting the results of this survey. First, because this study was only conducted in 4 eastern Taiwanese high schools, the research findings might not apply to all indigenous adolescents in Taiwan. Second, this study only revealed the presence of drug use, the type of drugs consumed remains unknown. Future research is warranted to collect more detailed data on this phenomenon. Third, the related information in this study was collected in the interview conducted by researchers and assistants, which may potentially lower the rate of sexual behaviors. Since in many Asian countries, sexual behaviors happened in an early age, or prior to marriage, are immoral. Finally, we conducted measurements at a single time point, which might not reflect long-term exposure to the sexual behavior among indigenous adolescents. Future studies using longitudinal study design would make the research more discursive.

## 5 Conclusions

This study identified the important predictors of indigenous Taiwanese adolescents’ sexual behavior, which comprise gender, having a current romantic partner, having multiple past romantic partners, and consuming alcohol or drugs prior to sex. This study found that the proportion of indigenous adolescents with self-reported sexual behavior was higher than that of the same age group within the general Taiwanese population, and that many of them with self-reported sexual behavior had used alcohol or drugs. Because drinking and drug use often lead to unsafe sexual behavior and are associated with sexual initiation, sex education programs for indigenous Taiwanese adolescents should begin in elementary school and should address the adverse effects of alcohol to prevent teenage pregnancy and sexually transmitted diseases.^[32]^ Such programs can also provide life skills and endorse abstinence from alcohol and drug use.^[14,15]^

We suggest that sex education for indigenous Taiwanese adolescents should be implemented in primary schools, with educational materials that address alcohol and drug use. Notably, such education should be developed with an understanding of indigenous cultures (e.g., drinking as a way to show joviality). It is crucial for young people to understand the impact that alcohol can have on sexual behavior and how it increases the occurrence of the risky sexual behaviors that can lead to unwanted pregnancy or sexually transmitted diseases.

## Author contributions

**Conceptualization:** Mei-Yu Yeh.

**Formal analysis:** Tao-Hsin Tung.

**Writing – original draft:** Li-Mei Lin.

**Writing – review & editing:** Mei-Yu Yeh.
